# Integrated machine learning based on cuproptosis and RNA methylation regulators to explore the molecular model of prostate cancer and provide novel insights to immunotherapy

**DOI:** 10.7150/jca.112843

**Published:** 2025-06-12

**Authors:** Junchao Wu, Wentian Wu, Jiaxuan Qin, Ziqi Chen, Rongfang Zhong, Xunkai Zhu, Jialin Meng, Peng Guo, Song Fan

**Affiliations:** 1 Department of Urology, The First Affiliated Hospital of Anhui Medical University, Hefei, 230022, China.; 2 Department of Urology, The Affiliated Jiangyin Hospital of Nantong University, Wuxi, 214499, China.; 3 Department of Oncology, The First Affiliated Hospital of Anhui Medical University, Hefei, 230022, China.; 4 Department of Urology, The University of Hong Kong-Shenzhen Hospital, Shenzhen, 518053, China.

**Keywords:** prostate cancer, cuproptosis, RNA methylation regulators

## Abstract

**Background:** As a highly prevalent tumor in males, prostate cancer (PCa) needs newly developed biomarkers to guide prognosis and treatment. However, few researches have elaborated on the function of cuproptosis-associated RNA methylation regulators (CARMRs).

**Methods:** We identified CARMRs based on single-sample gene set enrichment analysis and weighted gene co-expression network analyses. Subsequently, we performed 10 machine learning algorithms and 101 combinations of them to select the best model in TCGA, GSE70768, GSE70769, and DKFZ cohorts. Furthermore, we explored the potential function of CARMRs in the tumor microenvironment, immunotherapy, and tumor mutation burden (TMB). We validated the expression of the two genes with the largest regression coefficients using qRT-PCR.

**Results:** In our analysis, we successfully established a consensus prognostic model with 9 CARMRs based on the 101-machine learning framework. Furthermore, functional enrichment analysis revealed different metabolic and signaling pathways in the high- and low-risk groups. Notably, the high-risk group had a higher TMB, a lower level of immune infiltration, and a lower expression of immune checkpoints. Through drug sensitive analysis, we screened chemotherapy drugs suitable for different groups. Vitro experiments illustrated the high expression of C4orf48 and SLC26A1 in PCa compared with normal controls. The discovery was in concordance with bioinformatic analysis results.

**Conclusion:** A gene signature with 9 CARMRs was developed in our study, which served as biomarkers for PCa. This brings benefits in determining the prognosis of patients with PCa and guiding personalized treatment.

## 1. Introduction

Generally speaking, prostate cancer (PCa) has a mortality rate of 7.1%, ranking the third highest of all cancers in both sexes combined, according to the GLOBOCAN 2018 estimates of cancer incidence and mortality 1. Of note, PCa is the second most common malignancy and the most prevalent tumor in males in most countries, according to recent studies 2. From a meta-analysis, we figured that rational screening reduced the cancer incidence and mortality, reinforcing the importance of searching for biomarkers. As to the treatment modalities, except for the surgical castration, androgen deprivation therapy (ADT) remains be cornerstone of treatment for advanced PCa 3. Nevertheless, prolonged ADT can induce castration-resistant PCa in some patients, leading to a higher risk of distant metastasis and poorer recurrence-free survival (RFS) and increased risk for cardiovascular disease 3, 4. In this context, the search for appropriate therapeutic targets is of significant importance.

As an important cofactor in biological life processes, both defective and overloaded copper ions can cause abnormal biological effects. Based on the phenomenon described above, cuproptosis, as a novel mode of cell death, was proposed in 2022 5. Following this discovery, considerable evidence suggests that copper is significantly correlated with tumor-related biological processes, and hub genes of cuproptosis play a key role in tumor occurrence, metastasis, and escape 6-8. From a pan-cancer analysis, we also discovered that cuproptosis-related genes were negatively associated with the tumor microenvironment (TME) score, which demonstrated that these genes had correspondence with the reconstruction of TME and tumor immunosuppression 7. Meanwhile, previous studies had confirmed that RNA methylation is significantly associated with tumorigenesis and metastasis, and has the potential to serve as a biomarker for most cancers 9-12. Moreover, the crosstalk between both characteristics has been investigated in multiple cancers. Underlying a comprehensive review regarding the correlation between cuproptosis and RNA methylation regulators in hepatocellular carcinoma, we noticed that their crosstalk contributed to the exploration of valuable prognostic biomarkers, which could guide the application of targeted therapy 13. For instance, the dual role of METTL16 in participating in the m6A modification and inducing the process of cuproptosis was revealed, and a couple of cuproptosis-associated RNA methylation regulators (CARMRs) were further identified to construct risk models and characterize immune status 13-15. Additionally, YTHDC2 serves as a CARMRs to regulate the methylation and proliferation processes of colorectal cancer 16. The downregulation of it can facilitate the cuproptosis resistance. Similarly, ATG10, regulated by RNA m6A methylation, also plays a pivotal role in the cuproptosis and survival outcomes, emphasizing a promising tumor-regulatory function 17. Nevertheless, the investigation of CARMRs has been limited to the construction of a prognostic model, and the in-depth discussion of CARMRs in PCa has not been studied so far.

Therefore, in the current study, we integrated 10 machine learning algorithms and 101 algorithm combinations to construct a risk model in PCa patients based on CARMRs in the TCGA-PRAD dataset and validated our model in three independent public datasets. What's more, we performed vitro experiment to further validate the expression of CARMRs. And we subsequently explored the value of CARMRs in the aspects of prognosis, immune infiltration, and responses to treatments in PCa.

## 2. Materials and methods

### 2.1 Dataset acquisition and preprocessing

We acquired four independent public datasets from The Cancer Genome Atlas (TCGA), Deutsches Krebsforschungszentrum (DKFZ), and Gene Expression Omnibus (GEO) based on two criteria: (1) patients with available expression data and (2) comprehensive clinicopathological features, particularly RFS status and time 18. TCGA-PRAD was enrolled, including 490 patients with complete expression profile data and clinical information. The transcripts per million were computed and further log2(x+1) transformed for subsequent analysis, which reduced the dimensionality of the original expression data to achieve greater comparability. GSE70768 19 (n=111), GSE70769 19 (n=88), DKFZ (n=105) were employed to validate our signature. And the 'sva' package 20 was employed to remove potential cross-dataset batch effects along with the empirical Bayes framework. A summary of the clinicopathological parameters of the four datasets is shown in Table 1.

### 2.2. Single‑sample gene set enrichment analysis (ssGSEA) and Weighted gene co-expression network analysis (WGCNA)

Cuproptosis-related genes and RNA methylation regulators were collected from previous literature 5, 21. Defining R ≥ 0.7 and *P* ≤ 0.001 as the cutoff, Pearson correlation analysis was used to screen for CARMRs. We employed the R package “limma” 22 to screen out differentially expressed genes (DEGs) between normal and tumor samples (|logFC|> 1 and adjusted *P* value < 0.05).

ssGSEA is a widely used approach for quantifying the enrichment score of a particular gene set within a single sample 23. In our analysis, 25 CARMRs were utilized for ssGSEA to obtain the CARMRs score. The WGCNA analysis is a systematic biological approach that enables the characterization of gene association patterns between different samples to identify highly co-varying gene sets 24. Then, we constructed a co-expression network by WGCNA package. A clustering tree based on the eigengenes of modules calculated the dissimilarity of the module eigengenes. Associations between modules and the CARMRs score were assessed using Pearson's correlation analysis. Finally, we choose the intersection of the highest related model genes and differential genes between normal and tumor organization for subsequent analysis.

### 2.3. Construction of prognostic signature by integrative 101 machine learning approaches

Univariate Cox regression analysis was employed to screen for CARMRs affecting RFS in PCa patients. To minimize the risk of overfitting, we arranged 101 combinations of these 10 algorithms in the TCGA-PRAD training dataset for variable selection and model construction based on a tenfold cross-validation framework. The integrative algorithms included random survival forest (RSF), elastic network (Enet), Lasso, Ridge, stepwise Cox, CoxBoost, partial least squares regression for Cox (plsRcox), supervised principal components (SuperPC), generalised boosted regression modelling (GBM), and survival support vector machine (survival-SVM). Of note, some algorithms, such as CoxBoost, Lasso, RSF, and stepwise Cox, can screen for characters. Based on a ten-fold cross-validation framework and calculation of C-index, we could screen for the optimal model with the highest average C-index. Subsequently, the risk score for each PCa patient was calculated according to the following formula:



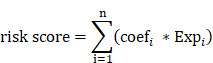



where coef_i_ and Exp_i_ represent the coefficient value and expression value of the corresponding gene, respectively. To assess the predictive accuracy and performance of the model, time-dependent receiver operating characteristic (ROC) curves and Kaplan-Meier (KM) curves were generated. Principal component analysis of independent prognostic genes was conducted.

### 2.4. Construction of the nomogram

We used the variables identified as independent prognostic factors for PCa as covariates to create a nomogram in the TCGA-PRAD cohort by employing the "regplot" package. This nomogram was designed to assess the recurrence rates of PCa patients at 1-, 3-, and 5-year. Additionally, we calculated the concordance index (C-index) and drawn the calibration curve to quantify the predictive effectiveness of the model by comparing it with other clinicopathological features.

### 2.5. Functional enrichment analysis and drug sensitivity analysis

Gene Ontology (GO) enrichment analysis and Kyoto Encyclopedia of Genes and Genomes (KEGG) pathway analysis were conducted to gain further insight into the potential biological mechanisms and pathways related to CARMRs utilizing “clusterProfiler” 25 and “org.Hs.eg.db”. Based on the curated databases of known molecular interactions and pathways, enriched pathways were selected using a threshold of adjusted *P* < 0.05. ssGSEA was performed to discover the potential functional signalling pathway and metabolic pathway in different groups. The hallmark gene set was downloaded from the Molecular Signatures Database, and 114 metabolic pathway gene sets were extracted from previous literature 26. As a common algorithm that can evaluate the enrichment extent of specific gene sets, the approach of ssGSEA was utilized by employing the “GSVA” R package to compute the actual score of diverse metabolic and signaling pathways in a single patient 27-29. We used the "Limma" package to calculate the differences in metabolism and pathways between the two risk subgroups, using a heat map to highlight the up- or down-regulation of each pathway. The “oncoPredict” package was utilized to calculate the half-maximal inhibitory concentration (IC50) values of common chemotherapy and targeted drugs for curing PCa. Experimental data on related drugs were downloaded from Genomics of Drug Sensitivity in Cancer (GDSC), the largest publicly available pharmacogenomics database currently.

### 2.6. Immune cell infiltration

Tumor growth and metastasis are affected not only by genetic variation and epigenetic regulation within tumor cells but also by the tumor microenvironment. Among the constituents of the tumor microenvironment, immune cells play a significant role in tumors. To explore the TME of PCa, we used the ssGSEA algorithm to evaluate the infiltration degree of 28 kinds of immune cells. The "IOBR" package and Wilcoxon test were conducted to verify the robustness and stability of ssGSEA results. Immune cells with no abundance in over half of the samples were excluded, and the differences in terms of immune cells across different risk groups were calculated using the “limma” package. Ultimately, the differentially distributed immune cells, identified by setting the threshold of *P* < 0.05, were vividly visualized by portraying a boxplot.

### 2.7. Quantitative real-time PCR (qRT-PCR)

The RNA extraction was performed using the Trizol reagent (Beijing ComWin Biotech Co., Ltd) from prostate normal and cancer cell lines RWPE-1, LNCaP, C42, PC3 (Wuhan Pricella Biotech Co., Ltd). For cDNA synthesis, reverse transcription was conducted using the TaKaRa (Dalian TaKaRa Biotech Co., Ltd) kit according to the manufacturer's instructions. GAP was employed as an internal reference gene to normalize relative expressions of lncRNA with the 2-ΔΔCT method. The specific primers in our study were as follows: C4orf48(forward: 5'-CGCCTTCGAGTTCATGCAG-3', reverse: 5'-CTGCAGCAGTAGGGTCTCC-3'); SCL26A1 (forward: 5'-CTGCGGGAGGAGATCCTAAG-3', reverse: 5'-GCACCACAGTGTAGTCGATG-3').

### 2.8. Statistical analysis

All the statistical analyses were carried out using R (version 4.3.0). Continuous data were analysed using an independent t-test or the Wilcoxon signed-rank test. The Fisher accuracy test was used to analyse the classified data. *P* < 0.05 was considered to indicate statistical significance.

## 3. Results

### 3.1. Identification of CARMRs

**Figure 1:**
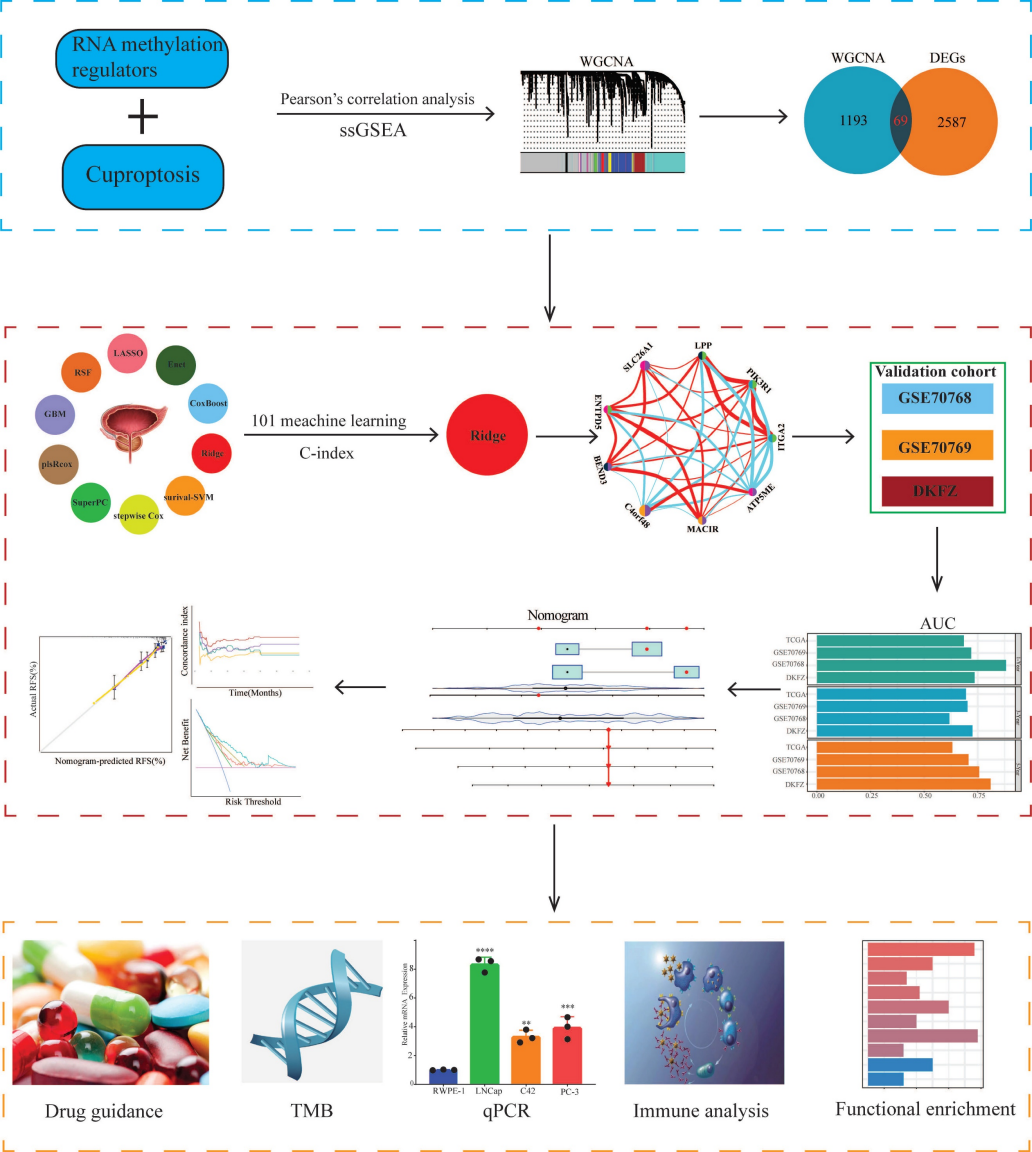
Flowchart of this research.

**Figure 2:**
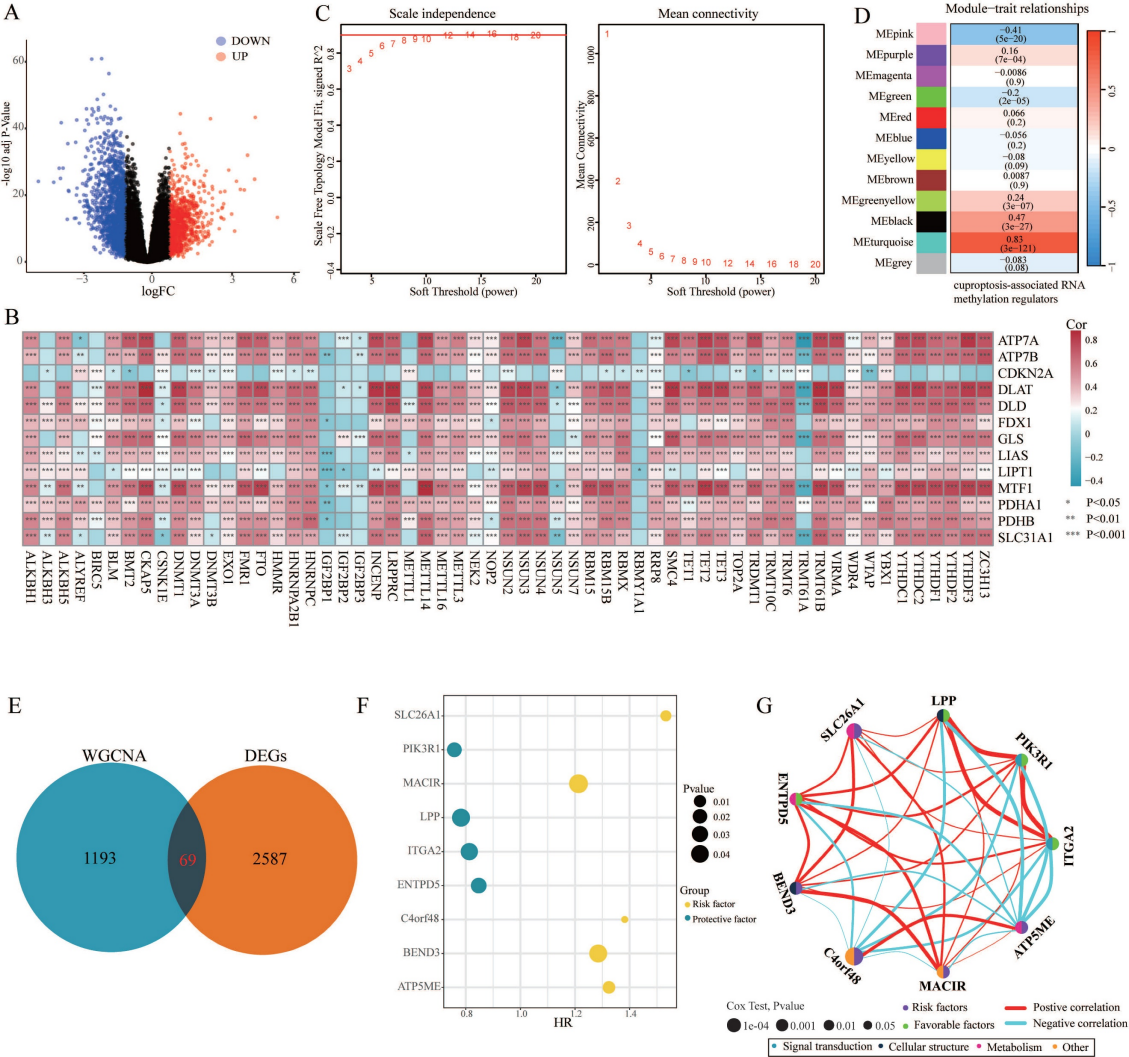
Identification of cuproptosis-associated RNA methylation regulators (CARMRs). (A) Volcano plot of differentially expressed genes (DEGs) in TCGA-PRAD. (B) Correlation associated between 13 cuproptosis genes and 56 RNA methylation regulatory genes. (C) Soft threshold (power =14) and scale-free topology fit index (R^2^ = 0.9016468). (D) Correlations of gene modules with CARMRs score feature. The values in the small cells of the graph represent the correlated coefficients between trait and module, as well as the corresponding statistically significant P-values. (E) Venn diagram of overlapping genes in DEGs and WGCNA. (F) Univariable Cox analysis of prognostic genes. (G) Gene interaction network diagram. *P < 0.05, **P < 0.01, ***P < 0.001.

The design ideas and process of our study were presented in Figure 1. A volcano plot of DEGs was illustrated in Figure 2A, and up-regulated genes were shown as red, while down-regulated genes were presented as blue. Meanwhile, we conducted Pearson's correlation analysis to figure out CARMRs from cuproptosis-related genes and RNA methylation regulators brought from previous literature. And we successfully identified 25 CARMRs preparing for further analysis (Figure 2B).

### 3.2. ssGSEA and WGCNA analysis

To further analyse the correlation between CARMRs and PCa, we conducted ssGSEA to calculate CARMRs scores in each sample from the TCGA-PRAD cohort. After that, CARMRs scores were utilized to perform WGCNA analysis, which could identify the modules correlated with CARMRs. In detail, when the soft threshold was set at 14 (no scale R^2 = 0.9016) (Figure 2C), 12 modules presented with different colours were identified in our analysis (Figure 2D). According to Figure 2D, we subsequently figured that the turquoise model was most significantly associated with CARMRs (cor = 0.83, *P* = 3e-27). Furthermore, we intersected the DEGs and the turquoise model genes (Figure 2E), and 69 CARMRs were subsequently selected for further research.

### 3.3. Construction of a CARMRs signature underlying integrative machine learning

**Figure 3:**
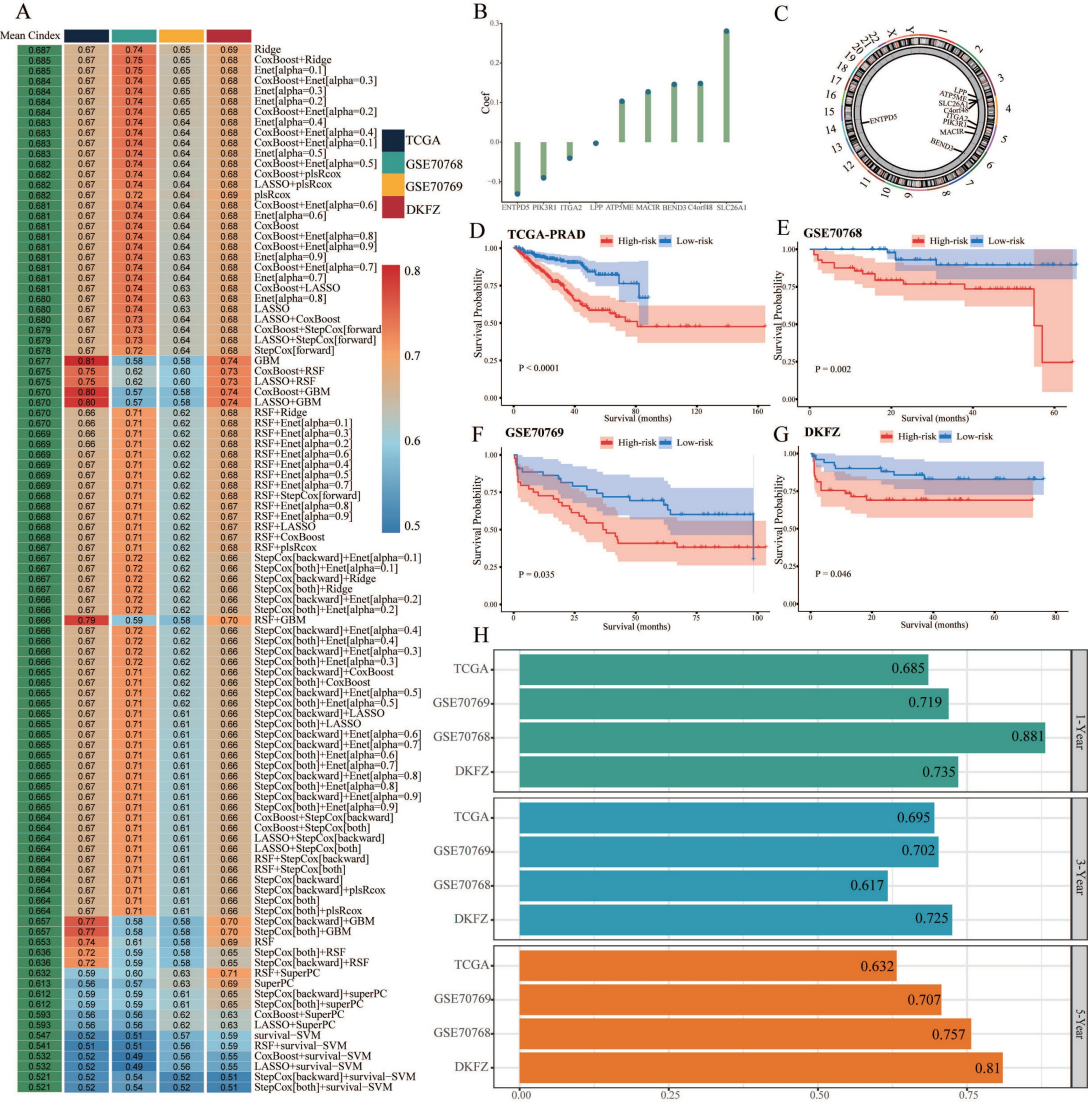
Construction and validation of the CARMRs via the 101 combinations of these 10 algorithms. (A) C-index of 101 kinds of prediction models calculated via a tenfold cross-validation framework in four cohorts. (B) Coefficients of nine genes were finally obtained in Ridge regression. (C) The position of nine genes in human chromosomes. (D-G) Kaplan-Meier curves of RFS according to the CARMRs in TCGA-PRAD, GSE70768, GSE70769, DKFZ cohorts. (H) The area under the curve for predicting RFS at 1-, 3-, and 5-year.

We conducted univariate Cox regression analysis to screen CARMRs affecting RFS in the TCGA-PRAD cohort, and finally obtained 14 prognostic genes. Due to the limitation of chip sequencing, five genes were not found in the microarray data, so we included 9 genes for follow-up analysis. A dot plot reflected the risk contribution of each factor according to the univariate Cox regression analysis (Figure 2F), and a correlation network indicated the interaction of 9 CARMRs and the underlying biological function correlated with PCa (Figure 2G). Whereafter, we performed an integrative 101 machine learning-based procedure with 9 prognostic CARMRs to develop a consensus CARMRs model (Figure 3A). Ultimately, according to the results, a prognostic model developed by Ridge showed optimal performance with the highest average C-index (0.687). The figure showed the distribution of coefficients of each gene (Figure 3B). The positions of CARMRs in the human chromosome complement were marked in Figure 3C.

What's more, we calculated the risk score for each cohort with the expression of 9 CARMRs and the coefficients we obtained above. The coefficients for each gene (ATP5ME, BEND3, C4orf48, MACIR, SLC26A1, ENTPD5, ITGA2, LPP, PIK3R1) were 0.103450158, 0.146147224, 0.148669511, 0.127294082, 0.280643611, -0.130781306, -0.040467373, -0.002685749, -0.090226076.

Therefore, we could categorize the patients in each cohort into high- and low-risk groups according to the median cut-off value of the risk score. Patients with higher risk scores demonstrated a worse prognosis when compared with those with lower risk scores in TCGA-PRAD, GSE70768, GSE70769, and DKFZ cohorts (*P* < 0.05, Figure 3D-3G).

### 3.4. Validation of the prognostic model

**Figure 4:**
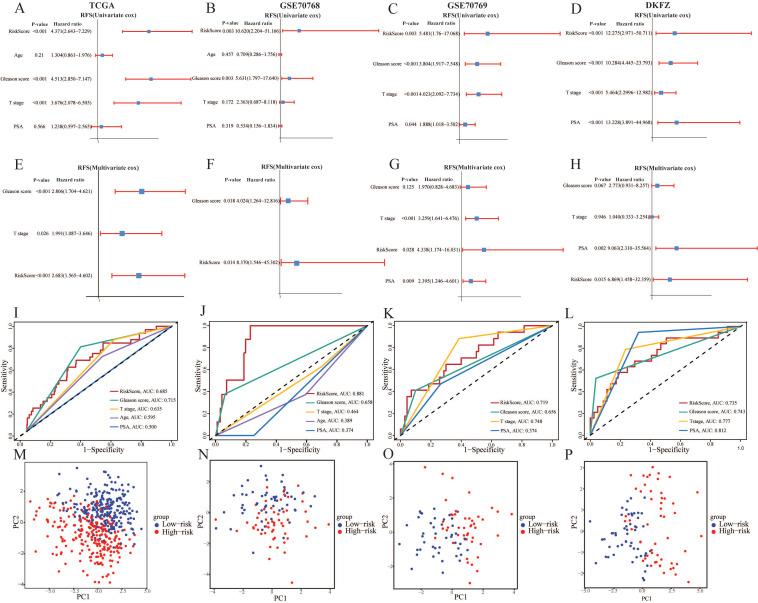
The predictive performance evaluation of CARMRs model. (A-D) Univariate Cox regression analysis of RFS in TCGA-PRAD, GSE70768, GSE70769, DKFZ cohort. (E-H) Multivariable Cox regression analysis of RFS in TCGA-PRAD, GSE70768, GSE70769, DKFZ cohort. (I-L) Time-dependent ROC analysis for predicting RFS of CARMRs and clinical characters. (M-P) Principal component analysis of four databases based on prognostic genes.

After identifying the prognostic genes, we performed TimeROC analysis to validate the predictive performance of the prognostic model. The area under the curve (AUC) of the prognostic model achieved 0.685, 0.695, and 0.632 for 1-year, 3-year, and 5-year intervals in TCGA-PRAD cohorts, and mostly achieved over 0.700 in the validation cohorts (Figure 3H). The results illustrated the superior predictive performance of the model. In addition, the univariate and multivariate Cox regression analyses demonstrated that the risk score was an independent risk factor to predict the RFS of PCa patients in all cohorts (*P* < 0.05, HR > 1) (Figure 4A-4H). Meanwhile, the clinical ROC curves were also conducted to reveal the efficacy of the risk score and other clinical characteristics. The results showed that risk score and Gleason score exhibited more favourable performance to predict the clinical outcome with higher AUC when compared with other clinicopathological parameters in all cohorts (Figure 4I-4L). PCA plots illustrating 9 prognostic CARMRs showed great performance in terms of distinguishing high- and low-risk groups in all databases (Figure 4M-4P).

### 3.5. Establishment and validation of nomogram based on clinical characters and CARMRs

**Figure 5:**
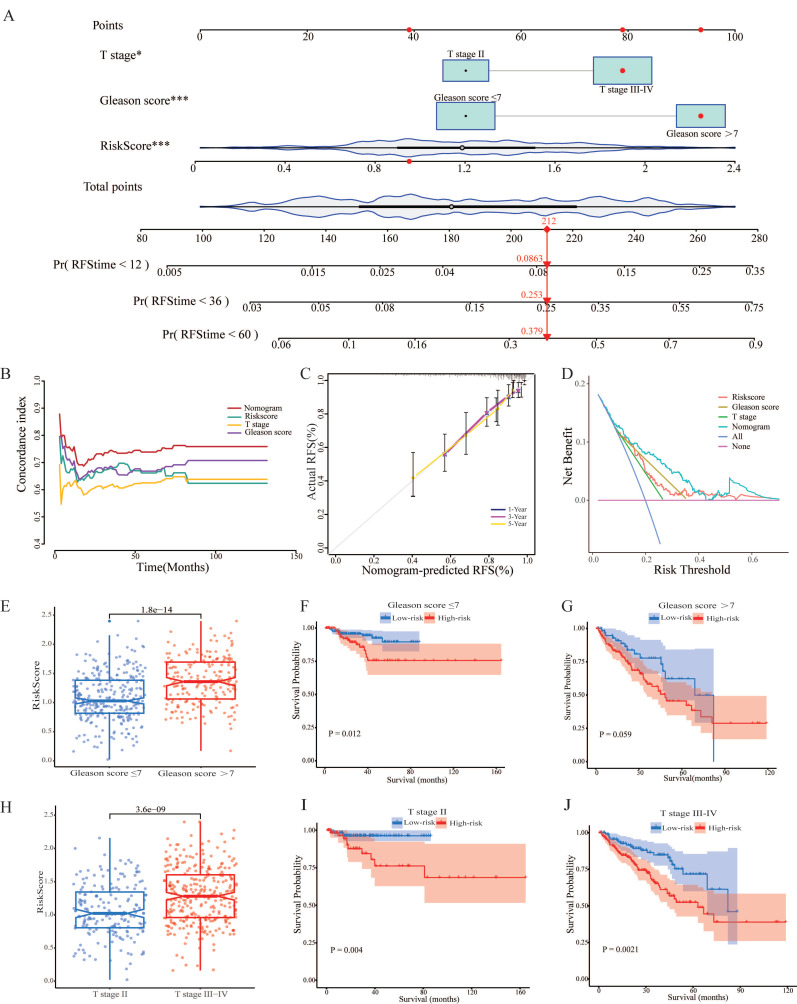
Establishment and validation of nomogram. (A) Construction of the nomogram based on the CARMRs, Gleason score, and T stage. (B) The comparison of the C-index between the nomogram and other characteristics. (C) Calibration curve of the nomogram for 1-, 3-, and 5-year RFS. (D) Decision curve analysis shows the net benefit to patients and the practicalities of our model. (E) Differences in risk score according to the Gleason score group and (H) T stage group. (F-G) Kaplan-Meier curves of RFS in the Gleason score group and (I-J) T stage group.

Having identified the risk score as a prognostic factor for our model, we also found that T stage and Gleason score were risk factors as well to predict the RFS, according to the results above. Therefore, we'd like to construct a predictive nomogram based on three risk factors (containing risk score, T stage, and Gleason score) in the TCGA-PRAD dataset to guide clinical diagnosis and prediction (Figure 5A). In addition, a higher C-index demonstrated the robust predictive power of the nomogram when it was compared with other clinicopathological parameters (Figure 5B). Simultaneously, the calibration curves illustrated perfect similarity between nomogram predictions and actual observations, supporting this view (Figure 5C). The decision curve analysis is able to evaluate the degree of net benefit to patients and the practicalities of our model. As shown in Figure 5D, it was indicated the nomogram had a higher net benefit than other clinical features, illustrating that the combination was suitable. Meanwhile, we found that the risk score remained a robust predictor in subgroups separated by the two clinical characteristics (Figure 5E, 5F, 5H, 5I, 5J). Regrettably, a significant difference couldn't be figured out between high- and low-risk groups in terms of survival probability when the Gleason score > 7 (Figure 5G). In a nutshell, we successfully constructed a nomogram to robustly predict the RSF of PCa patients and confirmed the efficacy of the nomogram.

### 3.6. Functional enrichment analysis of CARMRs and drug sensitive analysis

**Figure 6:**
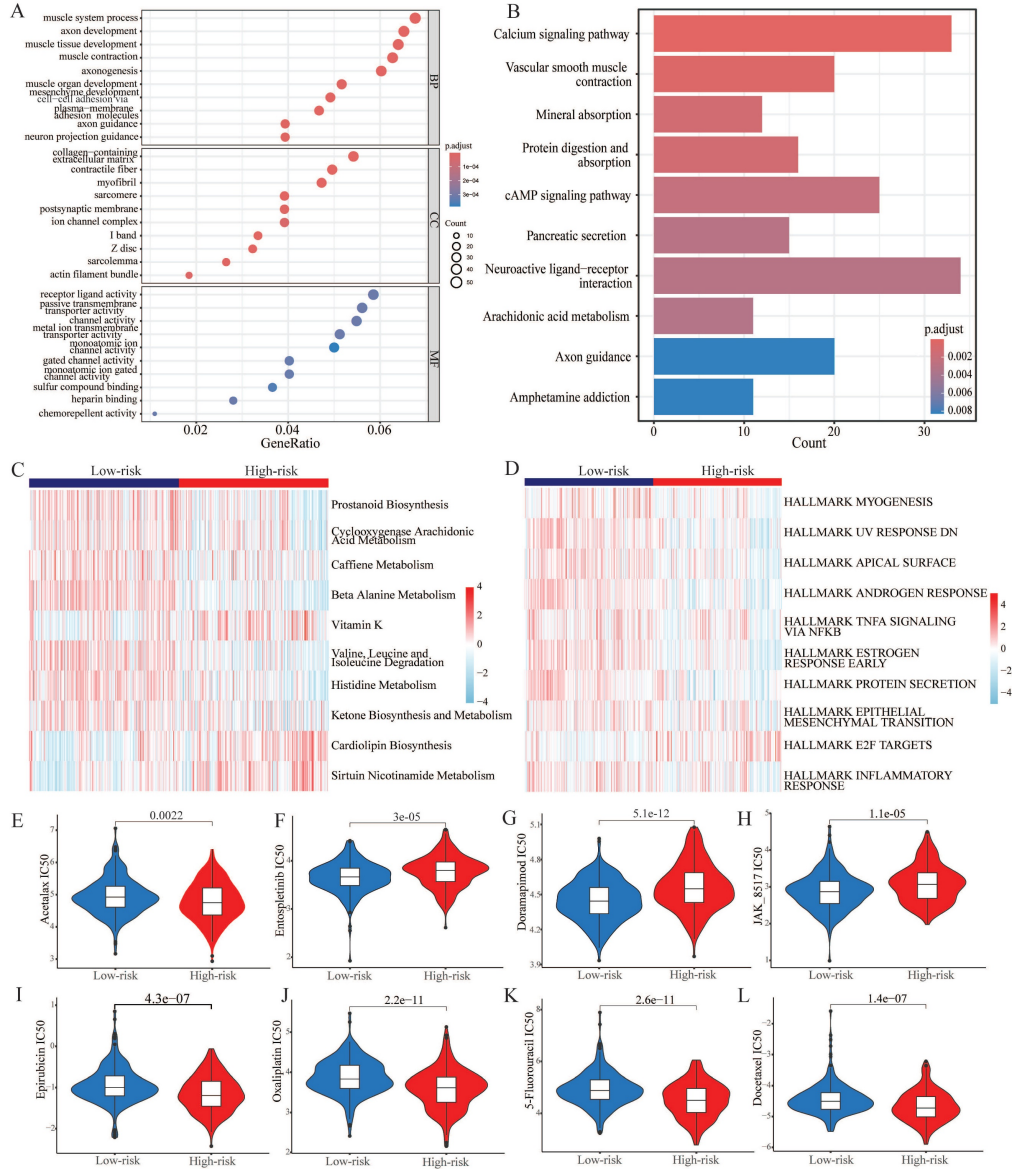
Biological function and ICI treatment of CARMRs for prostate cancer. (A) GO enrichment analysis. (B) KEGG enrichment analysis. (C) The ssGSEA score for CARMRs of metabolic pathway gene sets and (D) Hallmark gene sets. (E-L) Drug sensitivity analysis of Acetalax, Entospletinib, Doramapimod, JAK-8517, Epirubicin, Oxaliplatin, 5-Fluororacil, and Docetaxel.

In order to explore the potential biological function, we performed functional enrichment analysis on CARMRs for further research. According to the results of GO analysis, we identified that most of the CARMRs were enriched in axon development, axon genesis, muscle system process, muscle tissue development, muscle contraction, and muscle organ development in the biological processes (Figure 6A). In terms of cellular components, they were mainly enriched in collagen-containing extracellular matrix and contractile fiber. Meanwhile, a considerable number of CARMRs participated in channel activity, metal ion transmembrane transporter activity, monoatomic ion channel activity, passive transmembrane transporter activity, and receptor ligand activity in the molecular functions. In the KEGG pathway, the results indicated that CARMRs were enriched in axon guidance, calcium signalling pathway, cAMP signalling pathway, and neuroactive ligand-receptor interaction and vascular smooth muscle contraction (Figure 6B). Therefore, the results above demonstrated that CARMRs potentially participated in the vital activities of nerves and muscles. Furthermore, after comparing the enrichment levels of metabolism and signalling pathways between the high-risk and low-risk groups, we figured that patients in the high-risk group showed a decrease in prostaglandin synthesis and amino acid metabolism, but obtained an increase in lipid metabolism activity and enzyme synthesis (Figure 6C). Meanwhile, except for E2F targets signalling pathway, most of the signalling pathways (such as androgen response, epithelial-mesenchymal transition, and inflammatory response) were downgraded in the high-risk group as the heatmap showed (Figure 6D).

Utilizing the oncopredict algorithm, we compared the differences in terms of drug response of common chemotherapy and targeted therapy drugs for PCa between the high-risk and low-risk groups. And the IC50 values of Doramapimod, Entospletinib, and JAK-8517 were higher in the high-risk group, while the IC50 values of Acetalax, Docetaxel, Epirubicin, Oxaliplatin, and 5-Fluorouracil were higher in another group (Figure 6E-6L).

### 3.7. Immune infiltration analysis and tumor mutation burden

**Figure 7:**
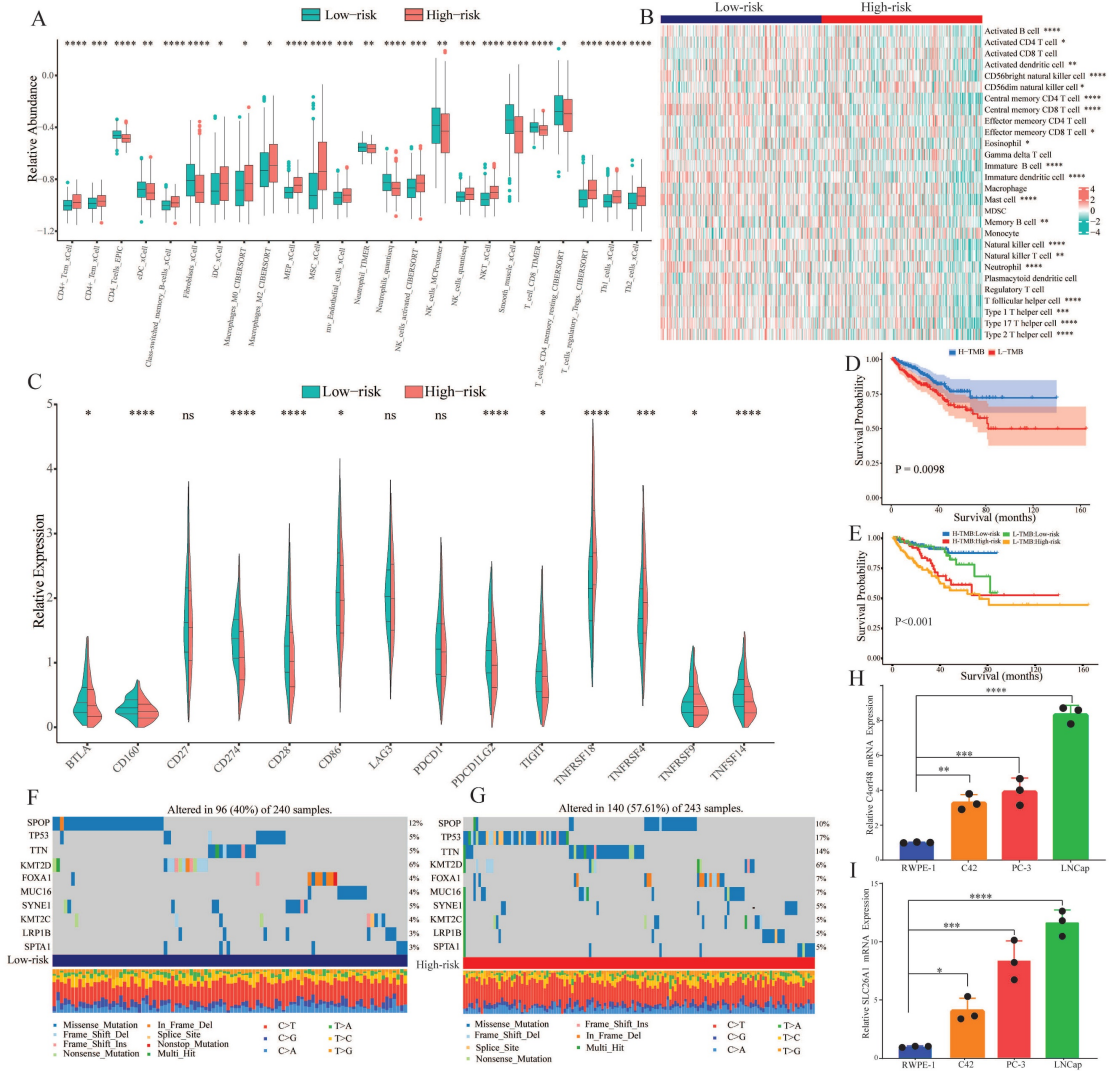
Tumor immune microenvironment and gene mutation. (A) The degree of immune cell infiltration in high and low risk groups was calculated by several algorithms. (B) Heatmap showing the differential abundance of 28 immune cells in the high and low risk groups. (C) Box plot revealing the expression level of common immune checkpoints between high and low risk groups. (D) Kaplan-Meier curves of the H-TMB and L-TMB groups. (E) Kaplan-Meier curves of the four subgroups separated by TMB and risk score. (F) Waterfall plot for the low-risk group and (G) high-risk group. (H) Expression of C4orf48 in normal prostate cells and prostate cancer cells. (I) Expression of SLC26A1 in normal prostate cells and prostate cancer cells. *P < 0.05, **P < 0.01, ***P < 0.001, ****P < 0.0001.

To explore the correlation of tumor immune microenvironment in different groups, we performed several algorithms, such as CIBERSORT, EPIC, TIMER, and xCell in the TCGA-PRAD dataset. The results showed that the abundance of most of the immune cells, especially memory cells and helper cells, was higher in the high-risk group (Figure 7A). While immune cells that played a killing role exhibited a decline in the high-risk group. According to the heatmap of immune infiltration (Figure 7B), we could explore that with the increase of risk score, the abundance of most immune cells would significantly reduce. Subsequently, we investigated the expression of common immune checkpoint-related genes in both groups. As shown in Figure 7C, most of the significant immune checkpoint-related genes were upregulated in the low-risk group, while the expression of two genes, including TNFRSF18 and TNFRSF4, increased in the other group.

Genomic mutation is an important risk factor for tumor genesis. To measure the levels of genomic mutation, we calculated TMB, which could reflect the total number of mutations in different subgroups. Subsequently, KM analysis was performed to explore the impact of TMB on the survival probability, and the results suggested that patients with higher TMB demonstrated a worse prognosis (Figure 7D). Underlying TMB and risk score, we separated the patients into four groups, and discovered that in spite of group, the high-risk group exhibited worse prognosis (Figure 7E). As the waterfall plot displayed, the top 10 gene mutations in the low-risk group had frequencies of 12%, 5%, 5%, 6%, 4%, 4%, 5%, 4%, 3%, and 3% (Figure 7F). Whereas in the high-risk group, the frequency of alterations was 10%, 17%, 14%, 6%, 7%, 7%, 5%, 5%, 5%, and 5% (Figure 7G).

### 3.8. Verification of the expression of CARMRs genes

We selected the two genes with the largest coefficient in Ridge regression analysis to verify their expression, C4orf48 and SLC26A1, which were measured in prostate normal or cancer cell lines by qPCR. As shown in Fig. 7H-7I, C4orf48 and SLC26A1 exhibited higher expression in prostate cancer cells compared to normal cells. These results implied that C4orf48 and SLC26A1 affect the occurrence of prostate cancer.

## 4. Discussion

In our research, we have attempted to develop a novel gene signature composed of 9 CARMRs utilizing several machine learning approaches. Ultimately, we identified ATP5ME, BEND3, C4orf48, ENTPD5, ITGA2, LPP, MACIR, PIK3R1, and SLC26A1 as the key prognostic genes to construct our model in the TCGA-PRAD cohort and verified their predictive performance in the validation cohorts. After confirming the gene signature was an independent risk factor in our analysis, we further explored the potential biological function and the correlation between prognostic genes and immune infiltration, drug response, and TMB. Finally, we chose the top two genes with the largest coefficients for experimental validation. As mentioned above, we identified that copper could cause tumor growth and spread by regulating the activity of protein kinase and has an elevated requirement in tumor tissue 30, 31. Therefore, the prognostic value of cuproptosis-related genes was investigated in a wide range of cancers 32-35. Despite the prognostic value of them was being confirmed, we still ignored the role of RNA methylation regulators, which are significantly associated with tumor metabolism and may be synergistic with cuproptosis. Consequently, we decided to study the role of CARMRs in the prognosis for patients with PCa.

Underlying CARMRs, the training and validation cohorts, we conducted machine learning utilizing 10 algorithms and 101 algorithm combinations, and finally, we constructed a prognostic model. As expected, the CARMRs signature was an independent risk factor of PCa, and the AUC in the TCGA-PRAD cohort proved the great performance of our prognostic model. Judging from the results, ATP5ME, BEND3, C4orf48, ENTPD5, ITGA2, LPP, MACIR, PIK3R1, SLC26A1 were identified as the prognostic genes.

ATP5ME can encode a subunit of mitochondrial ATP synthase. Studies on this term have shown that ATP5ME increases mitochondrial membrane potential and proliferation and decreases apoptosis and the levels of a variety of oxidative enzymes. Meanwhile, ATP5ME can downgrade the level of inflammatory immune cells in the microenvironment of tissue, contributing to alleviating the damage of inflammation 36. BEND3, serving as a transcription factor, is a crucial regulator for DNA methylation and bivalent promoters 37. T cells, which express BEND3, tend to release large amounts of inflammatory factors to recruit immune cells, such as activated B cells and neutrophils, and regulate the inflammatory response 38. Involving multiple signalling pathways, such as mTOR and AKT, ENTPD5 was discovered to contribute to the development, invasiveness, and chemotherapy response of PCa 39-41. ENTPD5 was discovered to be negatively correlated with the level of mast cells and natural killer cells, and these cells can inhibit tumorigenesis 42. Previous studies on ITGA2 have identified that ITGA2, which is enriched in exosomes of metastatic PCa, induces epithelial-mesenchymal transition and is supported by the results of functional enrichment analysis 43. ITGA2 contributed to the formation of immunosuppressive TMB by upgrading the level of Tregs, which had the capacity of hindering the function of CD4^+^ and CD8^+^ T cells 44. Macrophage Immunometabolism Regulator, also known as C5orf30, was discovered to regulate the migration and adhesion of cells and had an impact on the inflammatory responses that macrophages aroused 45, 46.

The heatmap of functional enrichment analysis has shown lipid and amino acid synthesis pathways were upgraded in the high-risk group, which confirms the view that in order to produce energy and avoid the citric acid cycle, PCa conducts proliferative activity through lipid consumption 47, 48. Meanwhile, the synthesis of some amino acids plays a key role in the tumor vital activities, causing inflammation stimulation and endothelial cell damage 49. The inflammation-related pathways' alteration indicated that metabolic reprogramming and the activation of inflammation potentially promote the formation of the tumor. Compared with dramatically upgraded metabolic processes, the high-risk group exhibited remarkably downgraded signaling pathways, encompassing epithelial cell activities (apical surface, androgen response, estrogen response, myogenesis, and protein secretion signaling pathways) and inflammatory processes (inflammatory response and TNF-α signaling pathways). Judging from previous articles, it's noteworthy for us to identify that 20-30% of PCa cases culminate in castration resistant outcomes 50-52, and the high-risk group, which tends to develop into a fatal form, shows a low response to androgen. On the other hand, the interaction involving tumor cells and muscle cells facilitates the process of epithelial-mesenchymal transition and expands the subpopulations of cancer cells with features characteristic of cancer stem-like cells, thereby hindering the normal muscle development, as well. E2F, as a dominant transcription factor, contributes pivotally to nucleotide biosynthesis and cell cycle by activating downstream targeted genes 53. Additionally, the E2F transcription factor exhibited strong correlation with chemotherapy resistance, higher Gleason score, advanced tumor stage, and tumor metastasis 54, 55. Therefore, a significant upregulation of E2F was observed in the high-risk group patients.

As shown in drug response analysis, Acetalax, Epirubicin, Oxaliplatin, 5-Fluorouracil, and Docetaxel reacted better in the high-risk group of PCa. Referring to some clinical research, we discovered that Epirubicin, Oxaliplatin, and 5-Fluorouracil achieved well-healing effects for the treatment of metastatic hormone-refractory prostate cancer, whether used as a single agent or in combination 56-59. Furthermore, previous literature revealed that Oxaliplatin had the ability to induce cell-cycle arrest and cell death, and had an effect to obstruct angiogenesis, which contributes to the formation and metastasis of PCa 60. Except for these common chemotherapeutic agents, Acetalax was detected as a potential therapeutic drug for PCa. Original research examined the function of Acetalax in terms of autophagy and mitochondrial dysfunction and discovered that it may inhibit tumor growth by the TNF-α signalling pathway, which was downgraded in the high-risk group 61, 62. In a word, we validated the value of common chemotherapeutic drugs and identified some prospective drugs in this section.

Abundant evidence indicates that TME may play a key role in the development, metastasis, and drug resistance of tumors and particularly contributes to the failure of immunotherapy. From single-cell and spatial transcriptomic analyses, it could be identified that immunosuppressive TME existed in PCa tissue 63. And the immunosuppression was associated with suppressive myeloid populations, exhausted T-cells, and high stromal angiogenic activity. To put it more clearly, the presence of low immune activation and low inflammatory response in PCa puts it in the category of “cold” tumors 64. Judging from the box plot, there a significantly lower levels of immune cells with the ability to trigger an inflammatory response and kill tumor cells (including CD4^+^ T cells, CD8^+^ T cells, NK cells) in the high-risk subgroup. On the contrary, Tregs, which could contribute to immune evasion, increased in this subgroup, and this phenomenon corroborates the viewpoint proposed in the previous articles 64, 65. Specifically, the enrichment extent of multiple immune cells was further validated in both risk subgroups by constructing the heatmap. Notably, a series of T cells, consisting of activated, central memory, helper T cells, and natural killer T cells, were observed to be remarkably lacking in the high-risk group, highlighting an immune cell deficiency and immunosuppression landscape. Previous studies also confirmed the potential inducing role of reduced and exhausted T cells in the onset and progression of diverse cancers 66-68.

To go further, we figured that the expression of common immune checkpoint-associated genes was significantly up-regulated in the low-risk group, which demonstrated that patients suffering from PCa in this group may respond better to the immune checkpoint inhibitors. Nevertheless, identifying the high expression of TNFRSF18 and TNFRSF4 promoted the exploration of immune checkpoint inhibitors for the high-risk group. Both of them belong to the TNF Receptor Superfamily Member, which serves as an engine for the activation of T cells and plays a key role in programmed cell death. As a novel immune checkpoint, TNFRSF18 is expressed on the surface of tumor cells and leads to immune escape. Recent phase 1 trials have further confirmed its anti-tumor activity and contribute to the application for various tumors 69. In addition, we perceived that compared with the low-risk group, the high-risk group had immunosuppressive TME, in which it had a significant decrease in T cells. Targeting TNFRSF4 and CTLA-4 pathways could arouse effective T cells to drive a robust anti-tumor response 70. What's more, Multiple studies have confirmed that other immune checkpoints have a strong correspond with the prognosis of PCa. In this case, the development of the CARMRs signature could determine the efficacy of immunotherapy for patients 71-73. Previous studies have shown that the tumorigenesis of PCa may be strongly associated with a variety of genetic mutations, which illustrate that TMB plays a key role in the tumor aggressiveness and therapy response of the related patients 2, 74. By examining the waterfall diagram, it is obvious to find that the TMB of the high-risk group is significantly higher than that of the low-risk group, which tends to lead to an increased probability of tumorigenesis and metastasis. The scatter plot had revealed the same results. Nevertheless, high TMB, particularly in the context of altered DNA repair, may lead to a better response to immune checkpoint inhibitors. This could explain why the high-TMB and low-risk subgroup obtained the best prognosis 75.

Moreover, our study has some limitations to solve. Though we successfully developed a CARMRs signature in the training and validation cohorts, there is still has necessity to validate our prognostic model in the actual clinical cohorts. Potential biological functions of 9 CARMRs need to be validated by more basic in vitro and in vivo experiments. Although we predicted the sensitivity of common drugs for PCa patients, prospective clinical drug trials are required to validate our predictions.

## 5. Conclusions

In conclusion, we identified a CARMRs gene signature in PCa by integrating 101 combinations of 10 algorithms to prevent overfitting. The construction of a prognostic model and nomogram held out the prospect for the diagnosis, personalized treatment, and prognostic evaluation of PCa. We not only certify the dependability of CARMRs' function in serving as therapeutic targets, but also assess the efficacy of related drugs based on the expression of CARMRs.

## Figures and Tables

**Table 1:** Summary of the clinicopathological parameters of the four cohorts.

Items	TCGA-PRAD(n=490)	GSE70768(n=111)	GEO70769(n=88)	DKFZ(n=105)
Age^a^				
≤60	220	48	—	105
>60	270	63	—	0
Pathological T satge				
T1+T2	187	34	46	68
T3+T4	303	77	42	37
Gleason score^b^				
≤7	287	102	74	91
>7	201	9	14	14
PSA^c^				
≤10	398	83	60	58
>10	44	27	26	47
Status				
Recurence free	398	92	44	81
Recurred	92	19	44	24

^a^Age information is missing in 88 patients from GSE70769 cohort. ^b^Gleason score information is missing in two patients from TCGA-PRAD cohort. ^c^PSA data are missing in 48 patients from TCGA-PRAD cohort, in one patient from GSE70768 cohort.
